# Construction by artificial intelligence and immunovalidation of hypoallergenic mite allergen Der f 36 vaccine

**DOI:** 10.3389/fimmu.2024.1325998

**Published:** 2024-03-27

**Authors:** Qiao-Zhi Qin, Jian Tang, Cai-Yun Wang, Zhi-Qiang Xu, Man Tian

**Affiliations:** ^1^ Department of Respiratory Medicine, Children’s Hospital of Nanjing Medical University, Nanjing, China; ^2^ Pediatric Department, Northern Jiangsu People’s Hospital, Yangzhou, China; ^3^ Department of Pharmacy, The Affiliated Cancer Hospital of Nanjing Medical University and Jiangsu Cancer Hospital and Jiangsu Institute of Cancer Research, Nanjing, China; ^4^ Research Division of Clinical Pharmacology, The First Affiliated Hospital of Nanjing Medical University, Nanjing, China; ^5^ National Vaccine Innovation Platform, Nanjing Medical University, Nanjing, China

**Keywords:** house dust mite, hypoallergenic vaccine, Der f 36, artificial intelligence, B cell epitopes

## Abstract

**Background:**

The house dust mite (HDM) is widely recognized as the most prevalent allergen in allergic diseases. Allergen-specific immunotherapy (AIT) has been successfully implemented in clinical treatment for HDM. Hypoallergenic B-cell epitope-based vaccine designed by artificial intelligence (AI) represents a significant progression of recombinant hypoallergenic allergen derivatives.

**Method:**

The three-dimensional protein structure of Der f 36 was constructed using Alphafold2. AI-based tools were employed to predict B-cell epitopes, which were subsequently verified through IgE-reaction testing. Hypoallergenic Der f 36 was then synthesized, expressed, and purified. The reduced allergenicity was assessed by enzyme-linked immunosorbent assay (ELISA), immunoblotting, and basophil activation test. T-cell response to hypoallergenic Der f 36 and Der f 36 was evaluated based on cytokine expression in the peripheral blood mononuclear cells (PBMCs) of patients. The immunogenicity was evaluated and compared through rabbit immunization with hypoallergenic Der f 36 and Der f 36, respectively. The inhibitory effect of the blocking IgG antibody on the specific IgE-binding activity and basophil activation of Der f 36 allergen was also examined.

**Results:**

The final selected non-allergic B-cell epitopes were 25–48, 57–67, 107–112, 142–151, and 176–184. Hypoallergenic Der f 36 showed significant reduction in IgE-binding activity. The competitive inhibition of IgE-binding to Der f 36 was investigated using the hypoallergenic Der f 36, and only 20% inhibition could be achieved, which is greatly reduced when compared with inhibition by Der f 36 (98%). The hypoallergenic Der f 36 exhibited a low basophil-stimulating ratio similar to that of the negative control, and it could induce an increasing level of IFN‐γ but not Th2 cytokines IL-5 and IL-13 in PBMCs. The vaccine-specific rabbit blocking IgG antibodies could inhibit the patients’ IgE binding and basophil stimulation activity of Derf 36.

**Conclusion:**

This study represents the first application of an AI strategy to facilitate the development of a B-cell epitope-based hypoallergenic Der f 36 vaccine, which may become a promising immunotherapy for HDM-allergic patients due to its reduced allergenicity and its high immunogenicity in inducing blocking of IgG.

## 1 Introduction

The house dust mite (HDM) has been widely recognized as a primary contributor to indoor aeroallergens, which is capable of eliciting IgE-mediated allergic diseases including allergic rhinitis (AR), asthma (AS), and atopic dermatitis (AD). Both AR and AS are the most common allergic diseases in children with an estimated prevalence range of 2%–25% and 3%–38%, respectively (1, 2). In China, HDM emerged as the leading allergen among children diagnosed with AR and/or asthma with approximately 60% of patients exhibiting sensitization to it (3). A previous study indicated that 74.5% of children diagnosed with AD were hypersensitive to HDM extracts as determined by skin prick test (4). The high prevalence of sensitization to HDM necessitates the development of effective therapeutics with substantial efficacy.

Allergen‐specific immunotherapy (AIT) is the only disease-modifying therapy for mite allergy (5). It was demonstrated to impede the progression of symptoms from mild to severe in children with mite allergy (6). The current vaccine used in AIT for mite allergy is still based on the natural HDM extracts. However, the quality of natural HDM extracts is limited (7–9). A previous study has reported significant variability among 10 commercially available HDM extracts, attributed to the absence of crucial allergens in the extract products and the varying IgE reactivity of different mite allergens (4), or which might contain several proteases, which can degrade proteins in HDM extracts (7).

The use of purified recombinant allergens for diagnosis enables the identification of specific allergens facilitating precision immunotherapy (10). However, native‐like recombinant allergens still retain IgE reactivity and T‐cell epitopes leading to immediate and late‐phase adverse reactions. Efficient approach might be achieved by recombinant hypoallergenic allergen derivatives. B-cell epitope-based hypoallergenic vaccines represent a further improvement for hypoallergenic derivatives of recombinant vaccine. These vaccines have the capability to induce a significant increase in allergen-specific IgG antibodies, which in turn can provide protection against allergic inflammation by inhibiting the reactivity of specific IgE and the activation of basophils in response to native-like allergens (11). The prediction of epitopes is a significant concern in the field of immunoinformatics and holds great practical implications for the development of epitope-based vaccines (12). Artificial intelligence (AI), defined as the utilization of computer algorithms and programs to mimic human intelligence (13), presents a promising avenue for enhancing the efficacy and success rate of traditional vaccine development. In the field of vaccine development, AI has been developed to forecast protein structure, predict B-cell epitopes (14, 15), determine class II major histocompatibility complex (MHC-II) epitopes, and facilitate the design of vaccines targeting the MHC-II immune peptidome (16, 17). Additionally, AI has been employed to identify potential biomarkers for seasonal flu vaccines (17), and analyze immune system response (18). Compared to the conventional ways, AI can be more accurate than animal models by taking human anatomy and physiology into consideration when simulating the effect of a new product (19). This would increase efficiency and reduce the cost for each drug.

Previous studies have highlighted the significance of groups 1, 2, and 23 in HDM as the most crucial allergens (20). Most hypoallergenic vaccine studies focused on the major allergens and individualized therapy for mite allergy (21). Der f 36 is a newly identified HDM allergen in recent years. It is a new allergen group without significant amino acid sequence similarity with known allergens. The IgE-binding frequency was determined as 42%–60% in France based on the recombinant Der f 36 and natural molecule (22), which made Der f 36 an important component for desensitization. However, there was no report focusing on the development of a hypoallergenic vaccine specifically targeting Der f 36.

In this research, we applied the AI strategy to facilitate the design of recombinant hypoallergenic B-cell epitope-based Der f 36 vaccine. The reduced allergenicity of the vaccine was characterized by enzyme-linked immunosorbent assay (ELISA), Western blot (WB), inhibition ELISA, basophil activation test, and peripheral blood mononuclear cell (PBMC) cytokine expression. The immunogenicity was evaluated through a rabbit immunization study. The IgG blocking of IgE-binding test and basophil activation test were also used to characterize the IgE inhibition ability of hypoallergic Der f 36 vaccine. We believe that the AI-driven designed hypoallergenic Der f 36 represents a promising strategy and vaccine candidate for immunotherapy of mite-allergic patients.

## 2 Method

### 2.1 Expression and purification of recombinant Der f 36

Der f 36 was already produced from the recombinant expression plasmid pET-28a (+) in *Escherichia coli* and stored at −80°C for the follow-up experiment. Briefly, the Der f 36 clone was inoculated in Luria–Bertani (LB) medium containing 50 μg/ml of kanamycin at 37°C and shaken at 200 rpm. When the optical density (OD) at 600 nm reached 0.6, isopropyl β-D-1-thiogalactopyranoside (IPTG) was added at a final concentration of 1 mM (shaking at 180 rpm at 37°C for another 4 h). The culture was harvested by centrifugation and disrupted by sonication for 20 min. The lysate was centrifuged at 12,000 × *g* for 10 min at 4°C, and the liquid supernatant was purified using HisTrap HP column (GE Healthcare, Uppsala, Sweden) and HiTrap Q HP column (GE Healthcare, Uppsala, Sweden) according to the manufacturer’s instructions. The concentration of endotoxin was determined by chromogenic method (details are presented in the **Supplementary Material**). Reverse-phase high-performance liquid chromatography (RP-HPLC) was used to determine the purity of Der f 36 (details are presented in the **Supplementary Material**).

### 2.2 Patients and samples

The Der f 36-positive serum was selected from 100 HDM positive serum in our preparatory work by ELISA (detailed procedures shown as below). HDM-positive sera were selected based on positive case history of HDM allergy and specific IgE to *Dermatophagoides farinae* > 0.35 kUA/L in serum, as determined by the ImmunoCAP System (Thermo Fisher Scientific/Phadia, Uppsala, Sweden). The non-allergic sera were used as the control group. HDM-positive sera with IgE antibodies specific to Der f 36 (n = 24) were selected for the assessment of hypoallergenic Der f 36. The study was approved by the ethics committee of the Children’s Hospital of Nanjing Medical University.

### 2.3 Construction of hypoallergenic B-cell epitope-based Der f 36 vaccine

#### 2.3.1 The tertiary structure construction and quality assessment of Der f 36

To design the hypoallergenic vaccine, the information of amino acid sequence of Der f 36 was retrieved from WHO/IUIS (http://www.allergen.org) and GenBank (protein ID: ATI08931.1). The amino acid sequence was submitted to Alphafold2 to build a three-dimensional (3D) model (23). PROCHECK (24) and ERRAT (25) in SAVES v6.0 serve (https://saves.mbi.ucla.edu/) was used to evaluate the model’s quality. Structural visualization was performed in PyMOL software (https://pymol.org/2/).

#### 2.3.2 Prediction of T-cell epitopes

T-cell epitopes were aimed to be avoided from the B-cell epitope-based hypoallergen to reduce the T-cell epitope-mediated late-phase side effects (26, 27). T-cell epitopes containing regions were predicted by TepiTool (http://tools.iedb.org/tepitool/) (28). The 26 most frequent human class II alleles from HLA-DR, HLA-DQ, and HLA-DP with a percentile rank ≤ 10 were set. The final T-cell epitope was selected from those containing two or more MHC-II alleles.

#### 2.3.3 Prediction of B-cell epitopes and allergenicity of synthesized B-cell epitope

Three methods, including AI and traditional computational prediction, were selected for linear B-cell epitope prediction. The 3D structure of Der f 36 was submit to the ElliPro server (http://tools.iedb.org/ellipro/) (29). To identify regions containing B-cell epitopes, the score was set at >0.5. Bepipred 2.0 (http://tools.iedb.org/bcell/) (30) and Graphbepi (http://bio-web1.nscc-gz.cn/app/graphbepi) were AI methods that were also used to predict B-cell epitopes (31). In Bepipred 2.0, the threshold was set at 0.5. The final linear B-cell epitopes were selected from the shared sequence from two or more prediction methods.

Conformational epitopes relied on the 3D structure of the protein. Conformational epitopes make essential contributions to allergenicity in inhalant allergens (32). Two AI methods, Seppa 3.0 (http://www.badd-cao.net/seppa3/index.html) (33) and SEMA (http://sema.airi.net) (34), were employed to predict the key residues contributed for discontinuous B-cell epitopes. The threshold of Seppa 3.0 was set at 0.064, and the SEMA value should be more than 1.1. All the results were integrated.

The final selected B-cell epitope peptides were synthesized by GenScript (Nanjing, China). Two milligrams of these epitope peptides was dissolved in 50 µl of ultrapure water, and a 2-μl sample was spotted on a nitrocellulose membrane to determine each epitope’s IgE-binding activity. Membranes were blocked with 1% BSA and incubated with 10 Der f 36-positive serum and one nonallergic sera (1:10 dilution) overnight at 4°C. The membranes were incubated with HRP-conjugated anti-human IgE antibody (1:5,000 dilution; KPL, MD, USA) and visualized by adding Immobilon™ Western HRP Substrate Luminol Reagent (Merck Millipore, MA, USA).

Moreover, the same method was used to detect these epitopes’ binding activity to human IgG4 except the membranes were finally incubated with mouse anti-human IgG4 antibody (1:5,000 dilution; Thermo Fisher Scientific, Massachusetts, USA).

#### 2.3.4 Design of hypoallergenic B-cell epitope-based Der f 36 vaccine

The PreS domain derived from hepatitis B virus was selected as a qualified carrier to integrate B-cell epitopes based on its immunogenicity and safety (35–37). The candidate fragments without IgE reactivity and without T-cell epitope regions were linked by KK linker at the N and C terminal of the PreS carrier (38, 39). The physicochemical properties were calculated using ProtParam (http://web.expasy.org/protparam/).

### 2.4 Expression and purification of constructed hypoallergenic Der f 36

The designed hypoallergenic B-cell vaccine gene combined with the C-terminal His-tag was synthesized by GENCEFE Biotech (Wuxi, China) and cloned into the pET-28a and transformed into *E. coli* strain BL21(DE3) (Tsingke, Beijing, China).

The expression of hypoallergenic Der f 36 was the same as Der f 36. The targeted protein was expressed as inclusion bodies and solubilized with 10 mM of Tris-HCL, pH 8.0, 100 mM of NaH_2_PO_3_ containing 8 M of urea, then purified by HisTrap™ HP affinity column. The protein was dialyzed into the refolding solution [0.5 M arginine, 1 mM of glutathione reduced (GSH), 0.1 mM of glutathione oxidized (GSSG), 50 mM of Tris, 0.5 M of NaCl, 5 mM of EDTA, 10% glycerol, pH 9.5] for 24 h at 4°C. The refolded protein was further purified by HiTrap™ SP HP cation exchange column (GE Healthcare, Uppsala, Sweden). The detailed elution condition is provided in the **Supporting Material**. The concentration of endotoxin was also determined. The purity of hypoallergenic Der f 36 was tested by RP-HPLC as described above. Anti-6×tis-tag antibody was used to verify the targeted protein by Western blot as described in the **Supporting Material**.

### 2.5 IgE reactivity of hypoallergenic Der f 36

#### 2.5.1 IgE reactivity analyzed by ELISA

The 96-well plates (Corning Costar, Maine, USA) were separately coated with 1 µg of hypoallergenic Der f 36 or Der f 36 in phosphate-buffered saline (PBS) per well then incubated at 4°C overnight. The plate was blocked with 1% BSA at 37°C for 2 h. The 24 IgE-positive sera, 100 µL/well (diluted in PBST PBS containing 0.1% Tween-20 1:10), were added and incubated at 37°C for 1 h. HRP-conjugated goat anti-human IgE (KPL, MD, USA) was added for 1 h, and chromogenic reaction was developed using TMB substrate (Beyotime, Shanghai, China) for 10 min. Then the reaction was stopped by 1 M of H_2_SO_4_, and the absorbance was measured at 450 nm using Multiskan GO (Thermo Fisher Scientific, MA, USA). The cut-off value was presented as mean OD value +3 standard deviations of control serum from healthy donors.

#### 2.5.2 IgE reactivity detected by Western blot

Both Der f 36 and hypoallergenic Der f 36 were electrophoresed on 12% SDS-PAGE and transferred onto 0.22-µm PVDF membranes (Merck Millipore, MA, USA). Membranes were blocked with 5% skimmed milk, then incubated with five representative sera from the set of Der f 36-reactive patients mentioned above and one negative control serum (1:10 dilution) at 4°C overnight. the membranes were detected with HRP-conjugated anti-human IgE. Visualization of the membranes was performed using the Tanon 5200 multi-imaging system (Tanon, Shanghai, China).

### 2.6 Inhibition of IgE binding to Der f 36

#### 2.6.1 IgE competition ELISA test

IgE competition ELISA test was used to analyze the ability of hypoallergenic Der f 36 to inhibit the IgE binding to Der f 36, which indicated the decreasing degree of IgE-binding activity of the designed vaccine. The plate was coated by Der f 36 (10 µg/ml) and diluted in PBS overnight at 4°C. Four samples were selected from the 24 positive sera to conduct a mixed serum pool diluted in PBST + 1% BSA (1:10). The mixed serum was pre-incubated with gradient concentration of Der f 36 and hypoallergenic Der f 36 at 4°C overnight, respectively. Each well was blocked with 1% BSA at 37°C for 2 h, then incubated by 100 μl of pre-incubated sera at 37°C for 1 h. The chromogenic reaction was the same as above. The formula for calculating inhibition rate was (OD450 uninhibited − OD450 inhibited)/(OD450 uninhibited − OD450 control) × 100%.

#### 2.6.2 IgG blocking of IgE-binding test

The immunogenicity of the hypoallergenic Der f 36 was evaluated, and its specific IgG was prepared by immunizing a rabbit using purified hypoallergenic Der f 36 to induce IgG (details are described in the **Supporting Material**). The specific IgG blocking of IgE binding to Der f 36 was also determined by ELISA. The ELISA plate wells were coated with Der f 36. After BSA incubation, 100 µl/well of 1:10 diluted rabbit serum was added and incubated for 1 h before reacting with the patients’ sera. The chromogenic reaction was the same as above.

### 2.7 Basophil activation test

Whole blood within donated by four volunteers who had Der f 36 specific-IgE was screened by dot blot and lysed with red blood cell lysis buffer (Fcmacs, Nanjing, China) and washed twice using PBS based on a previously published reported (40, 41). Basophils were stimulated with anti-human IgE antibody as a positive control, Der f 36, hypoallergenic Der f 36 (final concentration, 10 µg/ml), and PBS alone as a negative control at 37°C for 30 min. Then, pre-cooled PBS, including 2.5 mM EDTA, was used as ending solution, followed by centrifugation at 1,000 rpm for 5 min at 4°C, and the pellets were resuspended with 100 μl of the staining buffer containing 5 μl of FITC-conjugated anti-CD63 mAb (Biolegend, CA, USA) and 5 μl of PE-conjugated anti-CCR3 mAb (Biolegend, CA, USA) and followed by incubation on ice for 20 min. The expression of CD63 and CCR3 in basophil was evaluated by FACSCanto Plus and analyzed by FlowJo V10 software (Oregon, USA).

Inhibition of basophil activation by blocking IgG was further analyzed based on BAT. Briefly, the blocking IgG was purified from rabbit serum by Protein A affinity chromatography as described in the **Supporting Material**. The obtained blocking IgG (at 50 μg/ml) was mixed with Der f 36 for 30 min at 37°C, then the mixture or Der f 36 alone was added to the patients’ basophils for stimulating. Other steps were the same as described above.

### 2.8 PBMC cytokine expression

Fresh blood was donated from five Der f 36 allergic individuals. PBMCs were isolated by Ficoll density gradient centrifugation (Fcmacs Biotech China) and cultured with a cell count of 2 × 10^5^ in triplicate in 96-well plates (LabServ, Thermo Fisher Scientific, MA, USA) in 200 μl of serum-free Ultra Culture Medium (Gibco, Thermo Fisher Scientific, MA, USA) and stimulated with Der f 36, hypoallergenic Der f 36 (final concentration of 10 μg/ml) for 6 days at 37°C in 5% CO_2_ with a humidified atmosphere. The recombinant Der f 2 or PreS carrier was used as control protein. The supernatants were collected for cytokine ELISA using an ELISA kit of IL-5, 10, 13, and IFN-γ (Thermo Fisher Scientific, MA, USA).

### 2.9 Statistical analysis

The continuous variable was analyzed by paired sample *t*-test. The Mann–Whitney test was used for unpaired non-normal data. The paired non-normal data were analyzed by Wilcoxon test. A value of p < 0.05 was considered as statistically significant. Charts were completed by GraphPad Prism version 8.0 software (GraphPad Software, Inc., San Diego, CA, USA).

## 3 Results

### 3.1 Expression and purification of Der f 36

The recombinant Der f 36 was expressed and purified by Ni-NTA resin combined with HiTrap Q HP column. The major eluted fraction at 80 –90 ml was collected (**Supplementary Figure S1A**). The purified Der f 36 was migrated as a single band in SDS-PAGE with a molecular weight slightly below 25 kDa (**Supplementary Figure S1A**). The concentration of endotoxin was 0.11 EU/ml in Der f 36. The purity of Der f 36 assessed by RP-HPLC was 91.2% (**Supplementary Figure S1B**).

### 3.2 Construction of B-cell epitope-based hypoallergenic Der f 36

#### 3.2.1 3D modeling and evaluation of Der f 36 structure

**Figure 1:**
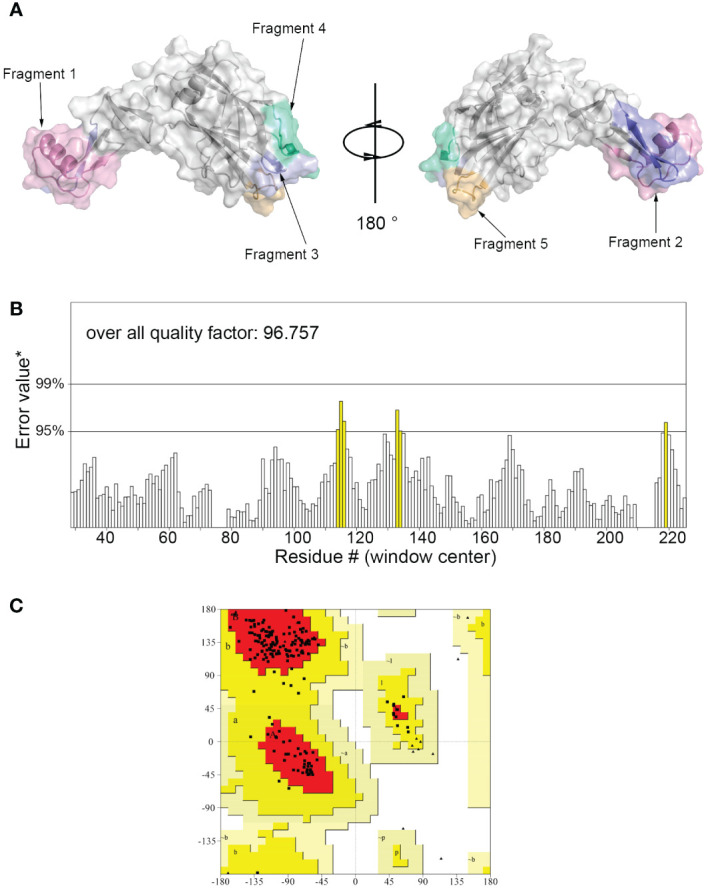
Three-dimensional (3D) modeling of Der f 36 and quality evaluation. **(A)** The 3D structure of Der f 36 allergen. The finally predicted linear B-cell epitopes are also displayed with different colors. **(B)** Validation of Der f 36 model by the ERRAT program (color figure online). **(C)** Validation of the Der f 36 model by Ramachandran plot. Residues in most favored regions (red); residues in allowed regions (yellow); residues in generally allowed regions (light yellow); residues in disallowed regions (white).

The 3D model of hypoallergenic Der f 36 protein was built by Alphafold2 (**Figure 1A**). The overall quality factor evaluated by ERRAT program is 96.757 (**Figure 1B**). None of the amino acid residues of Der f 36 were in disallowed regions of Ramachandran plot of tertiary structure (**Figure 1C**).

#### 3.2.2 Computational prediction of T-cell epitopes

TepiTool was used to predict the T-cell epitope of Der f 36. The regions of final predicted T-cell epitopes were 16–30, 21–35, 27–41, 33–47, 39–53, 44–58, 50–64, 55–69, 60–74, 67–81, 72–86, 77–91, 84–98, 91–105, 100–114, 105–119, 111–125, 116–130, 121–135, 129–143, 134–148, 139–153, 144–158, 149–163, 154–168, 167–181, 172–186, 181–195, 186–200, 193–207, 200–214, 205–219, 210–224, and 215–229. A detailed information of each epitope and binding alleles are presented in **Table 1**.

**Table 1:** The detailed information of predicted T-cell epitopes of Der f 36.

Peptide start	Peptide end	Peptide sequence
16	30	VAFFDNVHADSQAQE
21	35	NVHADSQAQEQCRQL
27	41	QAQEQCRQLHHVDID
33	47	RQLHHVDIDPSGTKF
39	53	DIDPSGTKFLNNNCR
44	58	GTKFLNNNCRLNCNI
50	64	NNCRLNCNIHGQIYG
55	69	NCNIHGQIYGHNINE
60	74	GQIYGHNINEGRTCM
67	81	INEGRTCMIGQTNYV
72	86	TCMIGQTNYVCQNGE
77	91	QTNYVCQNGECVGHN
84	98	NGECVGHNRQHVGHI
91	105	NRQHVGHIDIELISA
100	114	IELISASLYEKANAY
105	119	ASLYEKANAYATVCI
111	125	ANAYATVCIMNNSLP
116	130	TVCIMNNSLPISLPI
121	135	NNSLPISLPIQDRRN
129	143	PIQDRRNCISCSTHV
134	148	RNCISCSTHVKENTN
139	153	CSTHVKENTNYPVWN
144	158	KENTNYPVWNEVCVG
149	163	YPVWNEVCVGSSNYL
154	168	EVCVGSSNYLFVSDS
167	181	DSRVTTEVWDHHGSN
172	186	TEVWDHHGSNNNVFL
181	195	NNNVFLGGVTLTIDQ
186	200	LGGVTLTIDQLVNHG
193	207	IDQLVNHGDNHRQIN
200	214	GDNHRQINLAMAGGH
205	219	QINLAMAGGHNPGQL
210	224	MAGGHNPGQLSTRIT
215	229	NPGQLSTRITWTQRT

#### 3.2.3 Prediction of B-cell linear and conformational epitopes

We used three servers to predict the linear B-cell epitopes of Der f 36. According to the ElliPro server, seven linear epitopes were predicted as follows: residues 25–46, 92–97, 106–112, 141–164, 176–187, 198–207, and 212–218. Nine epitopes (residues 22–50, 64–70, 86–98, 107–110, 125–137, 142–151, 172–182, 196–204, and 213–221) were predicted by the Bepipred 2.0 server. Graphbepi showed eight epitopes (residues 25–48, 57–66, 68–72, 74–80, 122–135, 142–146, 177–184, and 209–216) (**Table 2**).

**Table 2:** The detailed information of predicted linear B-cell epitopes of Der f 36.

Server	Residues
Bepipred 2.0	22–50, 64–70, 86–98, 107–110, 125–137, 142–151, 172–182
ElliPro	25–46, 92–97, 106–112, 141–164, 176–187,198–207, 212–218
Graphbepi	25–48, 57–66, 68–72, 74–80, 122–135, 142–146, 177–184,209–216

**Figure 2:**
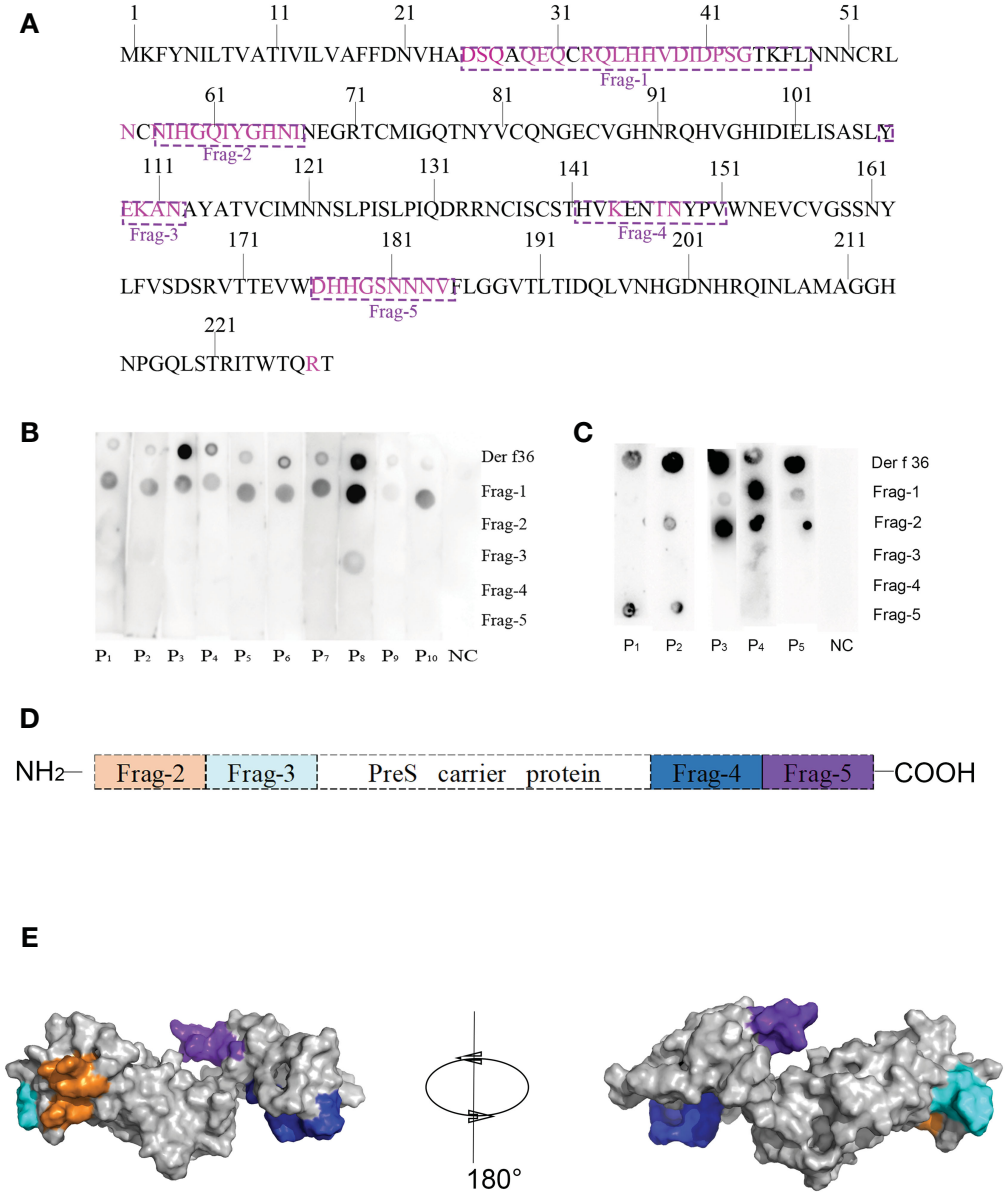
Design of B-cell epitope-based hypoallergenic Der f 36. **(A)** Distribution of B-cell epitopes in the amino acid sequence of Der f 36. The linear B-cell epitopes are boxed with dotted lines, and discontinuous B-cell epitopes are highlighted in purple. **(B)** Dot-blot assays were performed to detect each peptide’s IgE reactivity. **(C)** Dot-blot assays were performed to detect each peptide’s IgG4 reactivity. **(D)** Schematic representation of finally designed hypoallergenic Der f 36. Frag-2 to Frag-5 were used in hypoallergenic Der f 36 design. **(E)** The distribution of selected fragments on the 3D structure of the hypoallergenic Der f 36.

Forty-seven residues of conformational epitopes were predicted by Seppa 3.0 and SEMA. Most of them were included in the linear epitopes (**Table 3**). All of the linear epitopes and conformational epitopes were overlayed to get the final sequence. The final selected fragments containing B-cell epitopes without T-cell epitopes were colored in the 1D and 3D structure as follows: Fragment 1 (25–48), Fragment 2 (57–67), Fragment 3 (107–112), Fragment 4 (142–151), and Fragment 5 (176–184) (**Figures 1A**, **2A**).

**Table 3:** The prediction of conformational B-cell epitopes of Der f 36.

Residue number	Amino acid	Seppa 3.0 score	SEMA score
25	ASP	0.108	1.192
26	SER	0.113	2.160
27	GLN	0.096	1.977
29	GLN	0.125	2.201
30	GLU	0.111	2.540
31	GLN	0.111	1.961
33	ARG	0.140	2.570
34	GLN	0.133	2.878
35	LEU	0.116	2.095
36	HIS	0.127	2.347
37	HIS	0.152	2.886
38	VAL	0.163	2.731
39	ASP	0.157	2.609
40	ILE	0.161	2.121
41	ASP	0.152	2.322
42	PRO	0.144	2.246
43	SER	0.148	1.811
44	GLY	0.148	1.419
55	ASN	0.116	1.106
57	ASN	0.152	1.733
58	ILE	0.159	2.063
59	HIS	0.137	2.351
60	GLY	0.163	2.205
61	GLN	0.173	2.23
62	ILE	0.119	1.826
63	TYR	0.13	1.765
64	GLY	0.12	1.631
65	HIS	0.102	1.637
66	ASN	0.072	1.506
67	ILE	0.07	1.143
109	GLU	0.066	1.986
110	LYS	0.081	2.154
111	ALA	0.076	1.77
112	ASN	0.08	1.86
144	LYS	0.068	1.372
147	THR	0.076	1.739
148	ASN	0.078	1.616
176	ASP	0.067	1.82
177	HIS	0.071	2.481
178	HIS	0.071	2.848
179	GLY	0.092	2.604
180	SER	0.093	2.847
181	ASN	0.077	2.769
182	ASN	0.09	2.717
183	ASN	0.076	2.571
184	VAL	0.065	2.195
228	ARG	0.073	1.12

#### 3.2.4 Specific IgE and IgG4 reaction of synthesized B-cell epitope

Dot-blot assays were performed to detect IgE reactivity of the synthesized B-cell epitope fragments using sera from 10 Der f 36 allergic patients and one nonallergic serum. Fragment 1 showed 10 positive IgE-binding spots, Fragment 3 only weakly reacted with serum from patient 8, while others did not exhibit IgE reactivity (**Figure 2B**).

The top five sera with high IgE reactivity to Der f 36 in dot blot were selected to detect IgG4 reaction, and Fragments 1, 2, and 5 showed positive binding results (**Figure 2C**).

### 3.3 Design of hypoallergenic B-cell epitope-based Der f 36

To construct a B-cell epitope-based hypoallergenic Der f 36 vaccine, Fragment 1 with strong positive IgE reaction was deleted, and the other fragments (Fragments 2–5) were linked to the PreS carrier by the KK linker (**Figures 2D, E**). The ProtParam results showed that the vaccine has 217 amino acid residues with a molecular weight of 23 kDa and theoretical pI of 9.94. The total number of negatively charged residues (Asp + Glu) and positively charged residues (Arg + Lys) were 11 and 20, respectively. The grand average of hydropathicity (GRAVY) was −0.777.

### 3.4 Expression and purification of B-cell epitope-based hypoallergenic Der f 36

**Figure 3:**
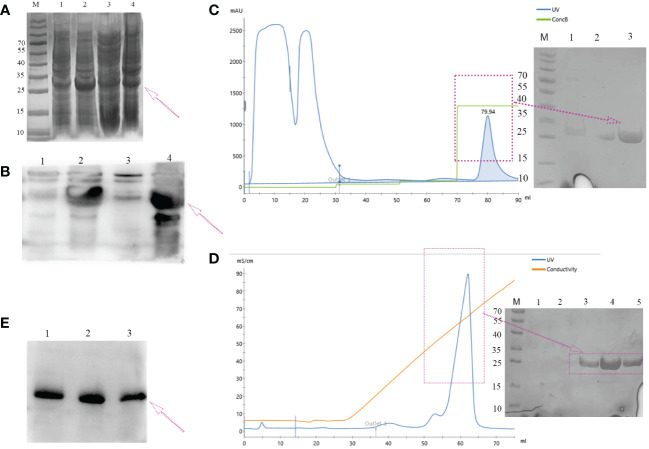
Expression and purification of hypoallergenic Der f 36 in (*E*) *coli*. **(A)** The recombinant protein resolved on 12% SDS-PAGE gel was visualized with Coomassie Blue G-250. Lane M, standard marker; lanes 1, 2 represent lysates of whole cells induced by 0 mM, 1 mM of IPTG, respectively; lane 3, the supernatant of cell lysate after ultrasonication; lane 4, the precipitants (inclusion bodies) of cell lysate after ultrasonication. **(B)** Non-induced (lane 1), induced (lane 2), supernatant (lane 3), and precipitated samples (lane 4) were verified by WB using anti-6*His antibodies. **(C)** Elution profile of hypoallergenic Der f 36 inclusion bodies on HisTrap™ HP affinity column. The fractions highlighted with an arrow were pooled for further purification. **(D)** The pooled fractions were further purified by loading onto a HiTrap SP HP column. The purified protein was highlighted with a purple box. **(E)** Final purified protein was verified by WB using anti-6*His antibodies.

The majority of the target proteins were located in inclusion bodies (**Figures 3A, B**, lane 4). The targeted protein was purified by nickel affinity chromatography under denaturing conditions containing 250 mM of imidazole (**Figure 3D**). The denaturant was gradually removed by dialysis, and the protein was refolded with redox refolding buffer. The folded protein was further purified by cation exchange chromatography and eluted as the main peak (**Figure 3C**). The molecular weight of the hypoallergenic Der f 36 was approximately 25 kDa by SDS-PAGE analysis. It was also verified using anti-6×His-tag antibodies by Western blot (**Figures 3B, E**). The concentration of endotoxin was 0.05 EU/mL in hypoallergenic Der f 36. The purity of hypoallergenic Der f 36 was 90.6% assessed using RP-HPLC (**Supplementary Figure S2**).

### 3.5 IgE reactivity of B-cell epitope-based hypoallergenic Der f 36

**Figure 4:**
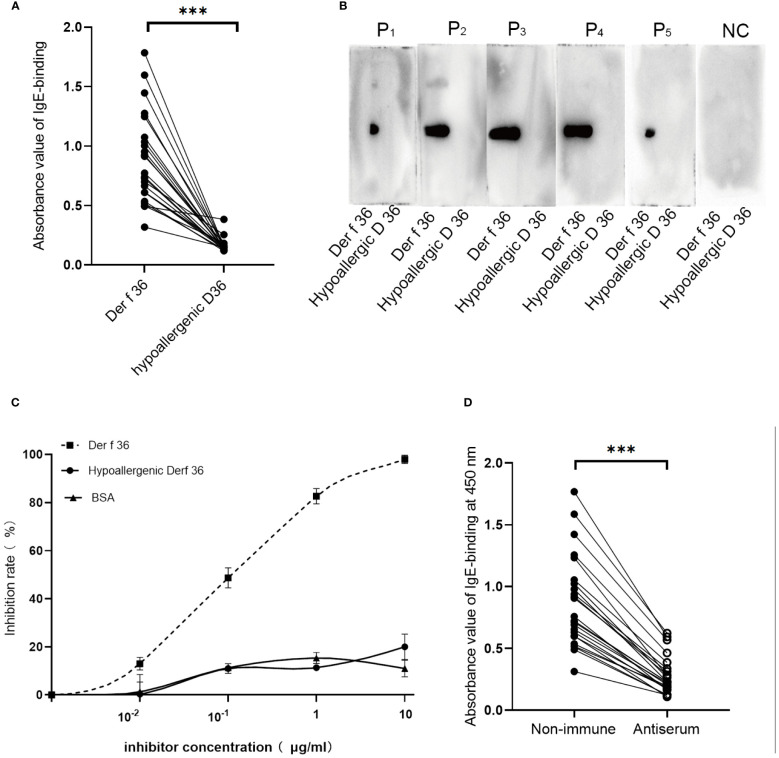
Sensitization and immunogenicity of hypoallergenic Der f 36. **(A)** Distinction of immunoreactivity to IgE between hypoallergenic Der f 36 and Der f 36 was assessed by ELISA. The result was analyzed using paired-sample t-test (^***^p *<* 0.001). **(B)** Confirmation of IgE reactivity by Western blot. The membranes were incubated with five positive serum samples and one negative control serum samples. **(C)** Competitive inhibition ELISA test with hypoallergenic Der f 36 and Der f 36. Pooled sera from four positive-reaction samples were used to conduct the inhibition ELISA with the increasing concentrations of the inhibitor. **(D)** Rabbit-specific IgG inhibits allergic patients’ IgE binding to Der f 36. The data were analyzed using paired-sample t-test (^***^p *<* 0.001).

ELISA and immunoblotting were performed to determine the allergenicity of the constructed vaccine. We compared the allergenicity of hypoallergenic Der f 36 with Der f 36 using 24 individual serum samples from Der f 36-allergic patients. All samples revealed a significant reduction in IgE-binding activity to hypoallergenic Der f 36 when compared to wild-type Der f 36 (4), (p *<* 0.001) (**Figure 4A**). The five representative allergic sera and one negative serum sample were analyzed using Western blot to confirm the result, in which only Der f 36 showed obvious IgE-binding bands, while no band was detected by hypoallergenic Der f 36 (**Figure 4B**).

### 3.6 IgE competition and inhibition test

A mixed serum pool of five high-positive-reaction sera was used to conduct a competition ELISA between the hypoallergenic Der f 36 and Der f 36. Only 20% of IgE binding to Der f 36 could be inhibited by hypoallergenic Der f 36 at 10 μg/ml, which is greatly reduced when compared with inhibition by Der f 36 (98%) (**Figure 4C**).

We then tested whether hypoallergenic Der f 36 IgG antibodies could inhibit the allergic patients’ IgE binding to Der f 36 in ELISA assays using 24 Der f 36-positive sera mentioned above. The blocking antibodies significantly inhibited the patients’ IgE binding to Der f 36 in each serum (p *<* 0.001) (**Figure 4D**). The inhibition rates of the blocking IgG were calculated between 57.0% and 81.7% (mean: 68.2%).

### 3.7 Basophil activation test

**Figure 5:**
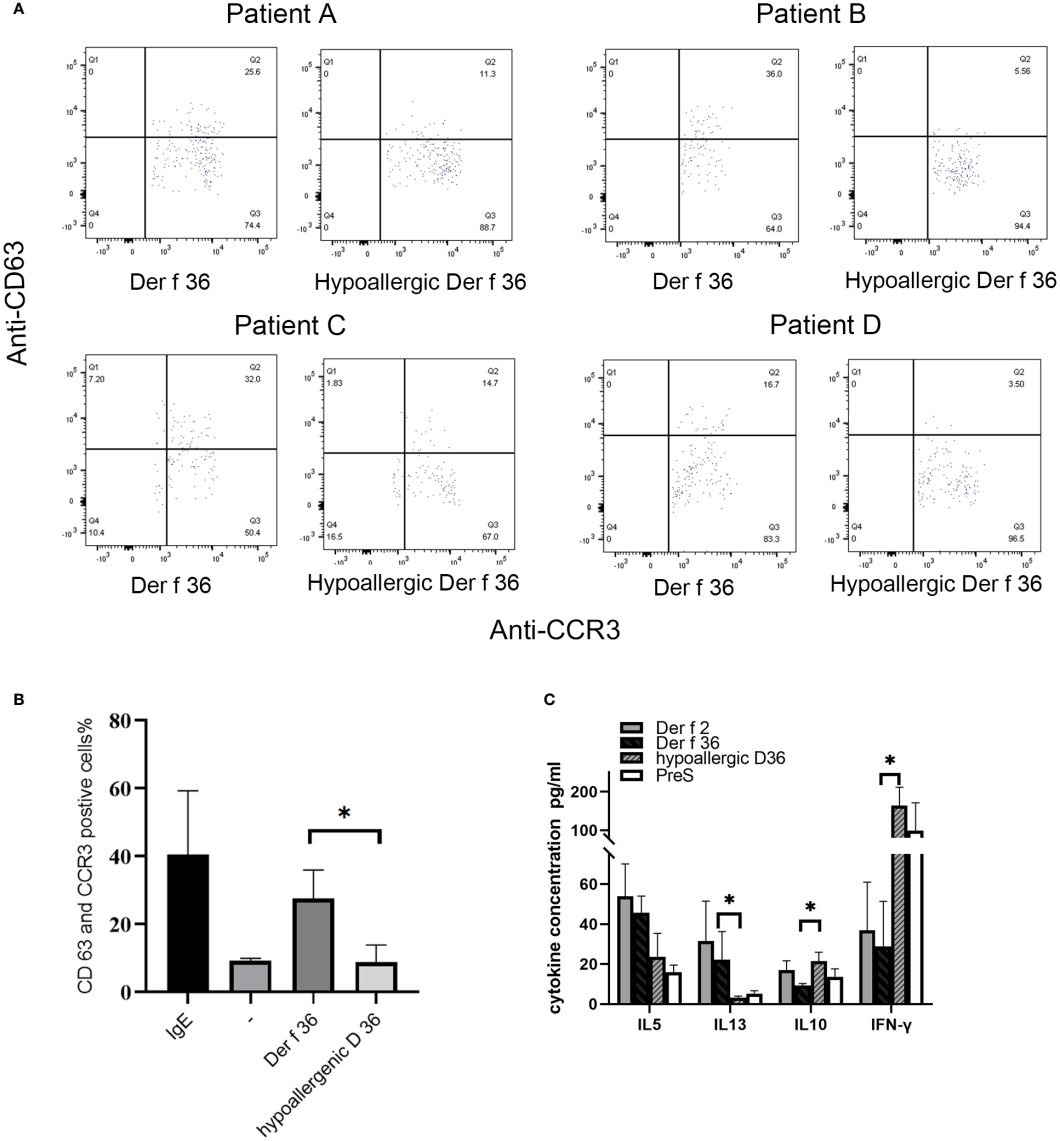
Induction of basophil activation by hypoallergenic Der f 36. **(A)** Basophil gating strategy and representative flow cytometry plots. **(B)** The basophils of Der f 36 allergic patients were stimulated with PBS, anti-IgE, hypoallergenic Der f 36, or Der f 36. The difference in stimulating rate between hypoallergenic Der f 36 and Der f 36 was analyzed using paired-sample t-test (^*^p *<* 0.05). **(C)** Comparison of PBMC cytokine expression between Der f 36 and hypo Der f 36. Stimulated with Der f 2, Der f 36, hypo Der f 36, and Pre S (final concentration of 10 μg/ml), supernatant fluids were collected for cytokine ELISA. Values were analyzed by the paired Wilcoxon test (*p < 0.05).

Basophil activation test was performed in 4 Der f 36-positive-reaction blood and a healthy donor. Proportions (%) of CD63-positive and CCR3-positive cells were detected to evaluate the basophil activation (**Figure 5A**). In comparison with Der f 36, the hypoallergenic Der f 36 causes less activation of basophils in the same cell sample, just as low as the negative control. The recombinant Der f 36 at 10 μg/ml induced an approximate 2.18- to 6.47-fold increase in the proportion (%) of CCR3 and CD63 double-positive cells compared with hypoallergenic Der f 36 (*p <* 0.05) (**Figure 5B**).

**Figure 6:**
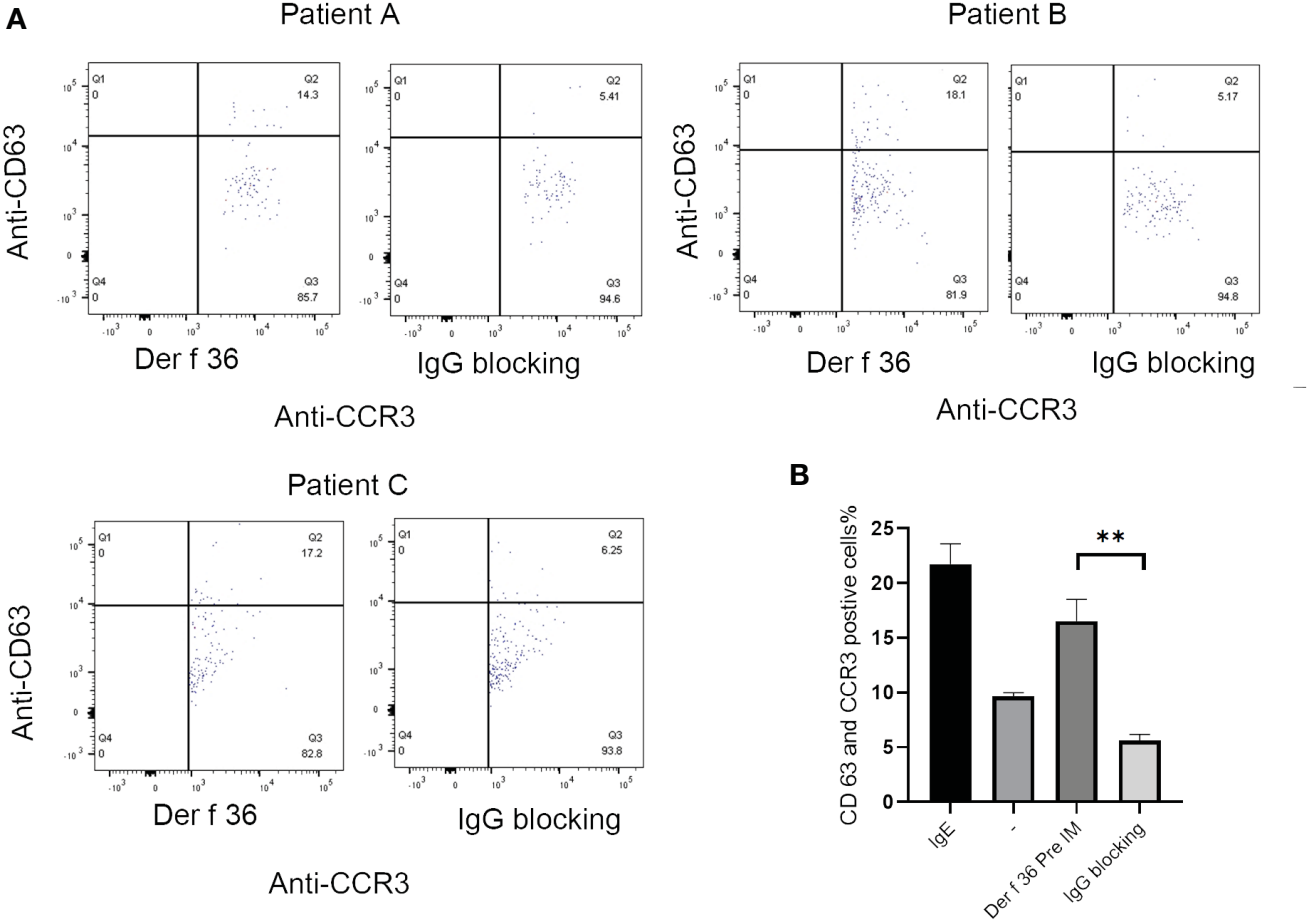
The inhibition of basophil activation of Der f 36 by specific IgG antibodies against hypoallergenic Der f 36. **(A)** The patients’ basophils pre-incubated with hypoallergenic Der f 36 IgG antibodies could lead to less activated basophils than non-blocking group. **(B)** The difference of stimulating rate between Der f 36 group and Der f 36 with IgG blocking group was analyzed by paired sample T-test (**P < 0.01).

The ability of blocking IgG to inhibit basophil activation was analyzed to confirm the protective efficacy of hypoallergenic Der f 36. Der f 36 pre-incubated with hypoallergenic Der f 36 IgG antibodies could lead to less activated basophils (5.17%–6.25%) when compared with non-blocking Der f 36 group (14.3%–18.1%) (*p <* 0.01) (**Figure 6**).

### 3.8 Cytokine responses of PBMC

The wild-type Der f 36 induced a higher level of Th2 cytokines IL‐5 (45.65 ± 8.34 pg/ml) and IL‐13 (22.27 ± 14 pg/ml) than hypoallergenic Der f 36 (23.68 ± 11.65 pg/ml for IL-5, p = 0.08; 3.23 ± 0.75 pg/ml for IL-13, p < 0.05). The hypoallergenic Der f 36 could induce a higher level of Th1 cytokine IFN‐γ (164 ± 47.88 pg/ml) than wild-type Der f 36 (28.92 ± 22.51 pg/ml) (p < 0.05). An increasing level of regulatory cytokine IL-10 was observed with the hypoallergenic Der f 36 (21.42 ± 4.48 pg/ml) than with wild-type Der f 36 (9.31 ± 0.91 pg/ml) (p < 0.05) (**Figure 5C**). Concurrently, Der f 2 induced the highest level of IL-5 (53.93 ± 16.18 pg/ml) and IL-13 (31.48 ± 20.03 pg/ml), but exhibited a lower level of IFN‐γ (36.91 ± 24.09 pg/ml) and IL-10 (16.98 ± 4.65 pg/ml) when compared with the hypoallergenic Der f 36 (**Figure 5C**). A lower level of Th2 cytokines was induced by the PreS protein since it is a non-allergenic carrier but could provide additional T-cell help activity and thus induce a high level of IFN-γ (**Figure 5C**).

## 4 Discussion

HDM is the most common allergen for allergic diseases. AIT was an effective treatment for HDM allergy (5). However, current AIT used in clinics has disadvantages such as the quality of the allergen preparation is uncontrollable and the risk of inducing adverse reactions (7–9). Recombinant and synthetic allergen derivatives have been applied to AIT to make it more safe and convenient. Clinical immunotherapy studies showed that the molecules contained IgE-reactive epitope or T-cell epitope, which may cause IgE-mediated or T-cell epitope-mediated late-phase side effects (26, 27). To improve the vaccine’s safety, non-allergic B-cell epitope-based hypoallergenic vaccine was developed (42). This type of hypoallergen is characterized by the lack of IgE reactivity without inducing any immediate side effects but preserving the ability to produce blocking IgG antibodies upon immunization (36, 43); for example, BM32 is a grass pollen allergy B-cell epitope-carrier vaccine, which has been reported to be safe and induces beneficial clinical effects in several clinical trials (36).

AI tools have been widely used in vaccine research that can facilitate a rational design and increase efficiency (19). In this study, we first applied AI strategy to facilitate B-cell epitope screening of Der f 36. For inhaled allergens, conformational epitopes appear to be the primary targets of IgE responses (32), so we integrated the predicted linear and conformational B-cell epitopes and excluded T-cell epitopes. The allergen’s candidate B-cell epitopes were further verified by serum-specific IgE reactions, and Fragment 1 showed a strong IgE reaction, which suggested that Fragment 1 made a great contribution on the allergenicity of Der f 36. Although Fragment 1 also showed positive IgG4 response, we still exclude it in the later construction of hypoallergenic vaccine for decreasing IgE-binding activity. The final selected Fragments of Der f 36 with low or without IgE reactivity were then fused to the Pres-carrier protein with the goal to reduce allergenicity while preserving high immunogenicity.

The constructed hypoallergenic Der f 36 was expressed in *E. coli* and finally purified as a high-purity vaccine. The majority of the target proteins were located in inclusion bodies. Only a weak His-tag-positive band could be observed in the supernatant. We deduced that the band with the same molecular weight as the supernatant may mainly be the endogenous protein of *E.coli*. Inclusion bodies also have advantages, such as easier isolation, resistance to proteolytic degradation, and very high amount of overexpressed protein (44). Such properties may also be suitable for the large-scale production of vaccines. The protein was migrated at approximately 25 kDa on SDS-PAGE, which was slightly higher than the calculated result and may be due to the additional 6×His-tag at the C-terminal of the expressed protein. The 6×His-tag facilitated the subsequent purification based on its affinity to Ni^2+^. Moreover, the theoretical pI of the hypoallergenic Der f 36 was calculated as 9.94, which indicated that it is a basic protein and would be positively charged under neutral or acidic conditions. Such property provided the possibility for further purification of the vaccine. Moreover, amebocyte lysate analysis showed that both Der f 36 and hypoallergenic Der f 36 were almost endotoxin-free molecules according to a previous study (45), which is crucial for animal experiment and PBMC cytokine expression.

This hypoallergenic Der f 36 showed reduction in IgE reactivity compared with Der f 36, as demonstrated by ELISA and immunoblotting. Competitive-inhibition ELISA also confirmed that the hypoallergenic Der f 36 lost most of its IgE-binding ability, and only 20% inhibition could be achieved, which is greatly reduced when compared with inhibition by Der f 36 (98%). Basophil activation test showed that the stimulating ratio of basophils in hypoallergenic Der f 36 was reduced as low as that of the negative control. Allergic individuals whose sera contain specific IgE have a bias in generating T-helper 2 (Th2) cytokines (46–49). IL-5 (50, 51) and IL-13 (52) are recognized as Th2 cytokines. IL-10 (53) and IFN-γ (54) have been reported to have a “protective” role in asthma. Hypoallergenic Der f 36 induced a lower level of the Th2 cytokines IL‐5 and IL‐13 than Der f 36. This indicated that the T-cell-mediated side effects of the vaccine may be decreased even though the difference of IL-5 was not statistically different. This is also the advantages of B-cell epitope vaccines due to the elimination of T-cell epitopes (26). We found that the hypoallergenic Der f 36 could induce a higher level of Th1 cytokine IFN‐γ than the wild-type Der f 36. We think the effect was attributed by PreS carrier based on its beneficial modulation of immune responses toward a Th1 phenotype according to previous studies (27, 55). Moreover, we also observed an increasing level of regulatory cytokine IL-10 by the hypoallergenic Der f 36 than wild-type Der f 36, which may be helpful for T-cell tolerance based on the immune-suppressive functions of IL-10 (56, 57). Moreover, as the controls, Der f 2 induced the highest levels of IL-5 and IL-13. This is similar to a previous study on carrier-bound Der p 2 peptide vaccine (58). The IFN‐γ and IL-10 induced by Der f 2 in our study was lower than Der p 2 in that study, which may be due to the different extent of sensitizations to Der f 2 from the patients in different studies. The PreS carrier is a non-allergenic carrier and induced very low levels of Th2 cytokines, but could provide additional T-cell help activity that is helpful for the production of blocking antibodies against the non-allergenic peptide. These results all confirmed that our constructed vaccine has low allergenicity and would be safer during therapy. To certify whether hypoallergenic Der f 36 could induce blocking IgG to inhibit IgE-binding activity of Der f 36, we used this vaccine to immunize rabbits and induced a high titer of IgG antibodies. This hypoallergenic Der f 36-specific IgG antibodies not only inhibited allergic patients’ IgE binding to Der f 36 but also inhibited the activation of basophils stimulated by Der f 36. All the evidence indicated that hypoallergenic Der f 36 has the potential to show protective efficacy as a qualified desensitization vaccine.

Individualized therapy using recombinant allergens has become popular (59). For HDM immunotherapy, several hypoallergenic allergen derivatives have been developed with reduced IgE reactivity (27, 60, 61). The application of AI has accelerated the clinical trial process and reduced the cost and time of vaccine development (19). Der f 36 is a newly identified allergen with a unique structure type. Its IgE-positive rate cannot be ignored. Overall, the hypoallergenic Der f 36 constructed using AI strategy represents a promising molecular vaccine candidate for immunotherapy of mite-allergic patients as well as an efficient paradigm strategy for the development of allergen vaccines.

## Data availability statement

The original contributions presented in the study are included in the article/**Supplementary Material**. Further inquiries can be directed to the corresponding authors.

## Ethics statement

The studies involving humans were approved by Children’s Hospital of Nanjing Medical University. The studies were conducted in accordance with the local legislation and institutional requirements. Written informed consent for participation in this study was provided by the participants’ legal guardians/next of kin. Ethical approval was not required for the study involving animals in accordance with the local legislation and institutional requirements because it was commercially available.

## Author contributions

Q-ZQ: Data curation, Formal analysis, Methodology, Project administration, Validation, Writing – original draft. JT: Data curation, Methodology, Software, Writing – original draft. C-YW: Data curation, Writing – original draft. Z-QX: Conceptualization, Investigation, Methodology, Writing – review & editing. MT: Writing – review & editing, Funding acquisition.
