# Supplementary material for: Construction by artificial intelligence and immunovalidation of hypoallergenic mite allergen Der f 36 vaccine
Source: Front Immunol. 2024 Mar 27;15:1325998. doi: 10.3389/fimmu.2024.1325998 (PMC11004385; doi:10.3389/fimmu.2024.1325998)
Supplement: Supplementary file 1 [file DataSheet_1.docx]

**Supporting material**

**Method**

1. Purification of recombinant hypoallergenic Der f 36 and Der f 36

According to the physicochemical analysis, the theoretical pI of Der f 36 is 6.37, this means it is an acidic protein, so we use anion exchange chromatography to perform protein purification. The samples were subjected to the HiTrap Q HP (5 mL) column (Cytiva, Uppsala, Sweden), and the protein was eluted with gradient increasing ion exchange, from solution A (20 mM Tris, pH 8.5) to solution B (20 mM Tris, 1 M NaCl, pH 8.5) at a flow rate of 5 mL/min. The eluent concentration increases from 0 to 50% in 10 min with 50 mL elution volume and then to 100% in 4 min with 20 mL elution volume. The negatively charged phosphoryl and carboxyl groups in endotoxin and the low isoelectric point (pI = 2) (1, 2) make it bound strongly to the anion exchange column under the buffer pH of 8.5, while the Der f 36 (pI = 6.37) bound weaker to the column than endotoxin, which make the Der f 36 eluted earlier than endotoxin and thus removed LPS from Der f 36.

Theoretical pI of hypoallergenic Der f 36 is 9.94, this means it is a basic protein, so we use cation exchange chromatography to perform protein purification. The samples were subjected to the HiTrap SP HP (5 mL) column (Cytiva, Uppsala, Sweden), from solution A (20 mM MES (2-Morpholinoethanesulphonic acid), pH 5.5) to solution B (20 mM MES, 1 M NaCl, pH 5.5) flow rates and total volume of elution were just as above. The basic property and high pI value of hypoallergenic Der f 36 make it bound strongly to the cation exchange column SP and eluted under high concentration of NaCl under the buffer pH of 5.5, while the endotoxin kept negatively charged and did not bind to the column, which make the removal of endotoxin from hypoallergenic Der f 36.

1. Determination of the purity by RP-HPLC.

The sample was injected into a C4 column (2.7 µm, 4.6 mm × 150 mm) (Halo, USA) which was pre-equilibrated with 85% solvent A (0.1 % v/v [trifluoroacetic acid](http://www-sciencedirect-com-s.webvpn.njmu.edu.cn:8118/topics/medicine-and-dentistry/trifluoroacetic-acid) in 100% water) and 15% solvent B (0.09% v/v trifluoroacetic acid in 95% acetonitrile) at a flow rate of 0.7 mL/min. The elution process involved a gradual increase in the proportion of solvent B from 15% to 100% over 30 minutes. The purity was calculated according to the integrated area of the corresponding peak at 280nm.

1. Anti-6×His-tag antibody Western blot

Both Der f 36 and hypoallergenic Der f 36 were electrophoresed on 12% SDS-PAGE and transferred onto 0.22 µm PVDF membranes (Merck Millipore, Massachusetts, USA). Membranes were blocked with 5% skimmed milk, then incubated with anti-6×His-tag Monoclonal antibody (1:5000 dilution) (Proteintech, Wuhan, China) at 4℃ overnight. the membranes were detected with Goat anti-mouse IgG (Proteintech, Wuhan, China). The visualization of the membranes was performed using the Tanon 5200 multi-imaging system (Tanon, Shanghai, China).

1. Determination of the amount of the remaining endotoxin

Chromogenic Lyophilized Amebocyte Lysate (LAL) endotoxin assay kit (Beyotime, shanghai, China) was used for detection of the remaining endotoxin in Der f 36 and hypoallergenic Der f 36. Briefly, endotoxin standards at 1 EU/mL, 0.5 EU/mL, 0.25 EU/mL, 0.125 EU/mL, 0 EU/mL (endotoxin-free water) and samples 10 μL were added into endotoxin-free 96-well plates (Labserv, Thermo Fisher Scientific, Massachusetts, USA), then endotoxin detection reagent was added 10 μL each well and incubated at 37℃ and avoid from light for 9 min. Ten μL of chromogenic substrate solution was added to each well and incubated at 37℃ for 6 min. Fifty μL buffer A, B, C were added into each well and mixed well. The absorbance of each reaction at 545 nm was analyzed by spectrophotometer. After analyzing the absorbance, we calculated endotoxin unit based on the standard curve obtained by standard solution.

1. Preparation and purification of rabbit specific IgG antibody

Rabbit specific antibody of hypoallergenic Der f 36 and Der f 36 were obtained by immunizing rabbit three times in monthly intervals with 200 μg of the purified protein. Titers of specific rabbit IgG for Der f 36, hypoallergic Der f 36 was determined by means of ELISA. Hypoallergenic protein induced IgG binding activity to Der f36 was also detected with ELISA. Rabbit’s serum was used as [primary antibody](javascript:;) and the secondary antibody was HRP-conjugated goat anti-rabbit IgG (Proteintech, Wuhan, China). Other steps have been mentioned in the article.

HiTrap rProtein A HP column was used for antibody purification. Rabbit ’s serum was diluted 1:1 in binding buffer (20 mM sodium phosphate, pH 7.0). After equilibration of the column with 10 volumes of binding buffer, the diluted serum was applied on the column. Then the column was washed with binding buffer until the absorbance reaches the baseline. The rabbit IgG was eluted with 100 mM glycine-HCl (pH 3.0) and immediately neutralized with 1 M Tris-HCl (pH 9.0).

The PreS carrier protein was expressed in *E.coli* by the same method of hypoallergenic Der f 36. The recombinant was expressed as refolded as previously described(3).

**Result**

**Reactivity of rabbit specific IgG antibodies to Der f 36.**

Rabbit serum obtained after immunization with hypoallergenic Der f 36 were tested for IgG reactivity and titers to Der f 36 by ELISA. Both of he two immunized rabbit sera have strong reactivity to its immune protein (**Fig. S4 A, B**), hypoallergenic Der f 36 IgG antibodies also showed IgG reactivity to Der f 36 (**Fig. S4 C**). Specific IgG was purified by HiTrap rProtein A HP column and identified by SDS-PAGE (**Fig. S4 D**).

**Information of IgE diagnosis of the 5 highly reactive sera.**

All of the 5 sera had a sIgE level to *Dermatophagoides farina* equal or greater than 5 (Table 1).

Table 1. Information of IgE diagnosis of the 5 highly reactive sera.

| Patients’ No | Age(year) | Gender | symptom | tIgE (kUA/L) | sIgE to *Dermatophagoides farina* (kUA/L) | sIgE Grade |
| --- | --- | --- | --- | --- | --- | --- |
| 1 | 5 | F | AS | 1285 | >100 | 6 |
| 2 | 7 | M | AR | 872 | 74 | 5 |
| 3 | 8 | M | AR+AS | 247 | >100 | 6 |
| 4 | 5 | M | AR | 172 | 87.6 | 5 |
| 5 | 7 | F | AR | 274 | 100 | 5 |

M: male; F:female; AS: allergic asthma; AR: allergic rhinitis; sIgE Grade: Grade 3 (3.5-17.5 kUA/L), Grade 4 (17.5-50 kUA/L), Grade 5 (50-100 kUA/L), Grade 6 (>100 kUA/L)

**Concentration of endotoxin in purified protein**

Following the instructions, we draw a standard curve using a standard endotoxin and calculate according to the formula. The concentration of endotoxin in Der f 36 is 0.11 EU/mL and the concentration of endotoxin in Der f 36 is 0.05 EU/mL

**SDS-PAGE analysis of recombinant PreS carrier and Der f 2**

The purified protein resolved on 12% SDS-PAGE gel was visualized with Coomassie Blue G-250. The molecular weight of PreS and Der f 2 were approximately 18 kDa and 15 kDa by SDS-PAGE, respectively (**Fig. S5**).


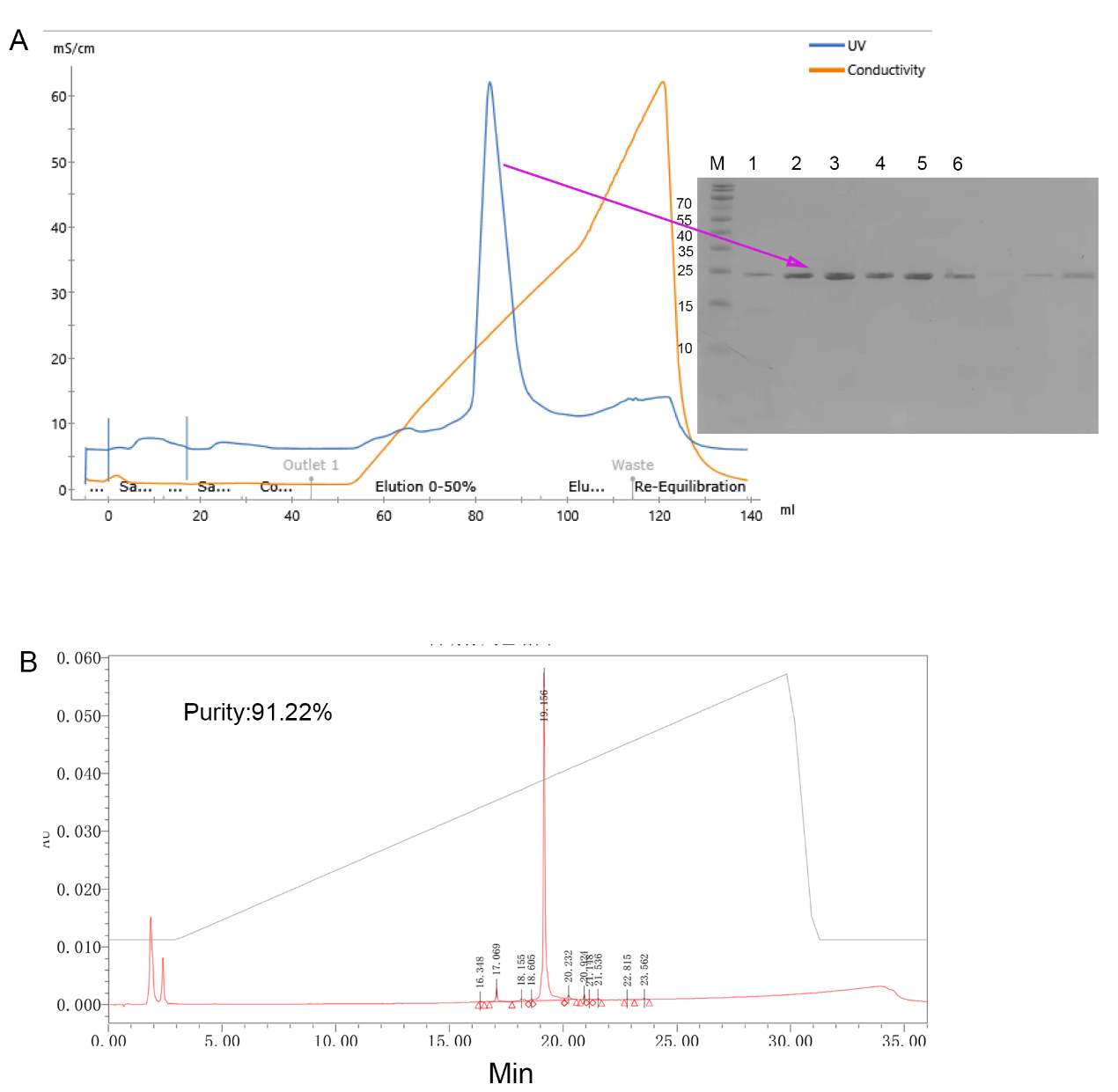


**Figure S1:** Expression and purification of Der f 36 **A** The pooled fractions were final purified by loading onto a HiTrap Q HP column. Lane 2 represented the purified protein.

**B** The purity of Der f 36 detected by RP-HPLC**.**


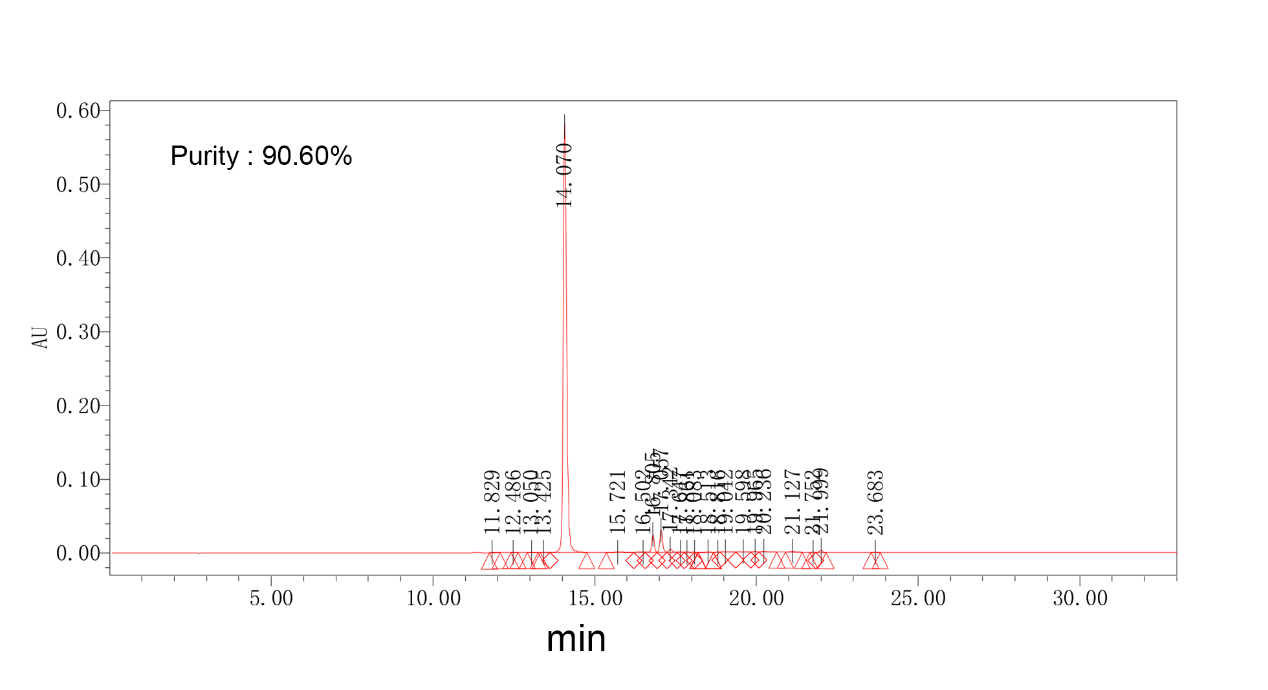


**Figure S2:** Purity of hypoallergenic Der f 36 **A** The purity of Der f 36 detected by P-HPLC**.** **B** Purity calculation of Der f 36


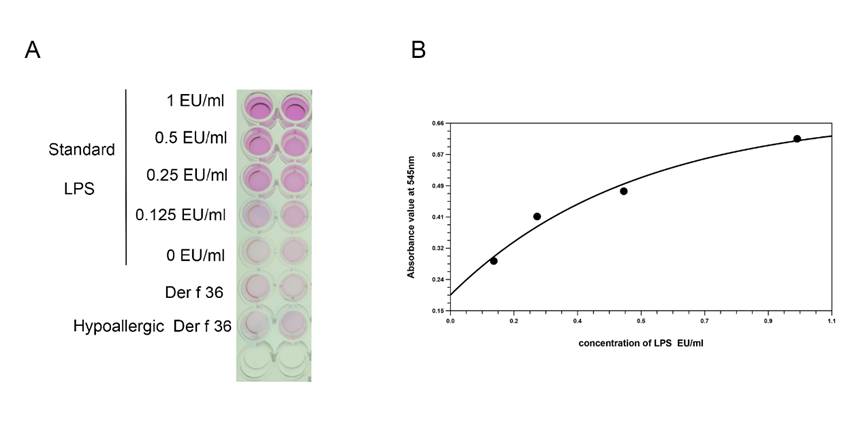


**Figure S3:** Determination of endotoxin in purified protein. the chromogenic method for detecting endotoxin by compare target protein with standard LPS. B. standard curve of LPS.


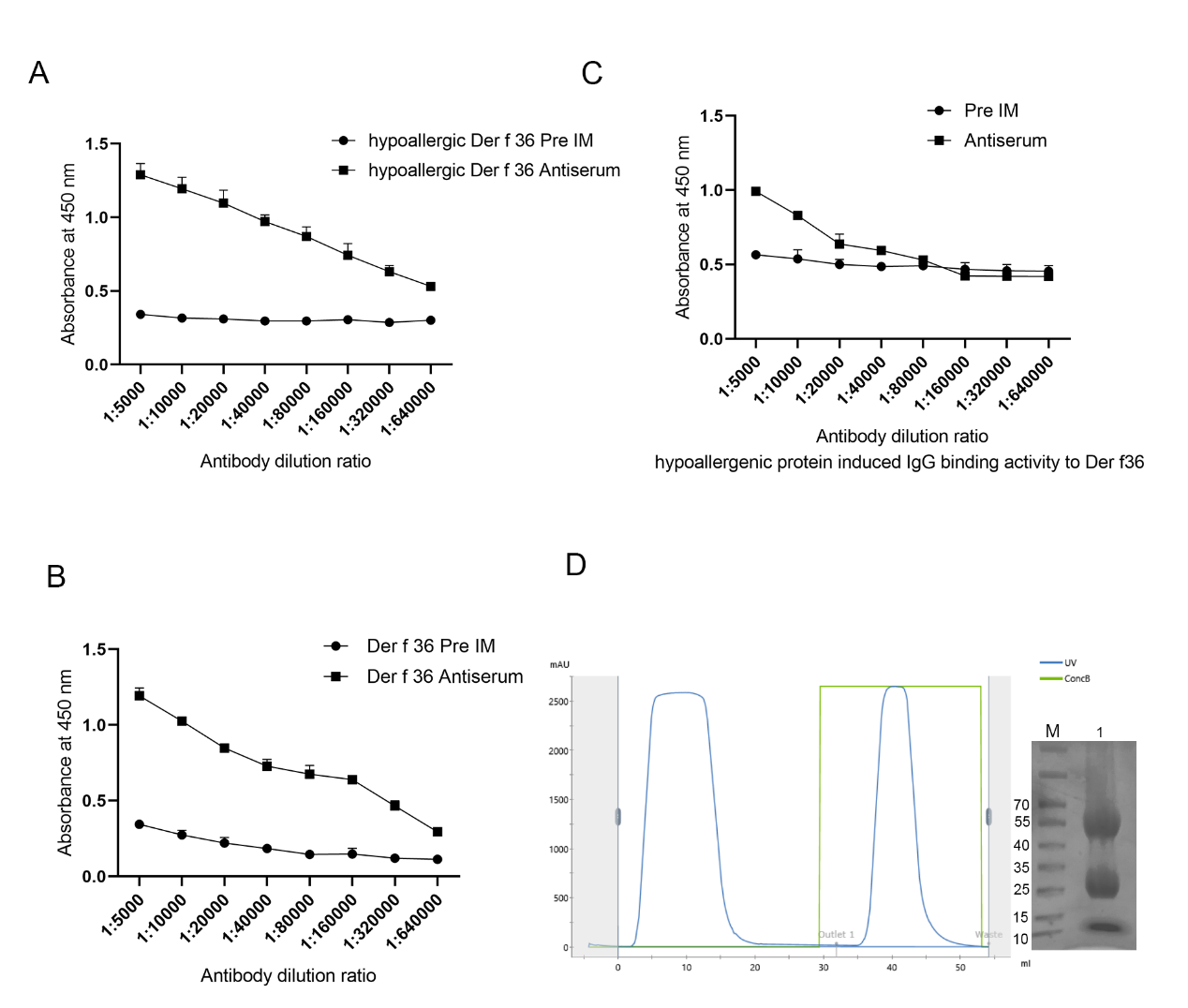


**Figure S4:** Assessment and purification of hypoallergenic Der f 36 induced rabbit specific IgG. **A** Rabbit serum was tested for IgG titers to hypoallergic Der f 36. **B** Rabbit serum was tested for IgG reactivity to Der f 36. **C** Hypoallergenic protein induced IgG binding activity to Der f 36. **D** Rabbit sera was purified by HiTrap rProtein A HP column.


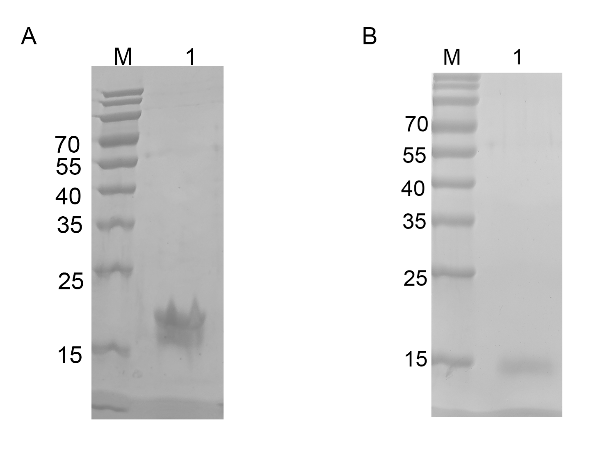


**Figure S5:** **A** Final purified PreS carrier protein **B** Final purified recombinant Der f 2

**References**：

1. Gorbet MB and Sefton MV. Endotoxin: the uninvited guest. *Biomaterials* (2005) 26(34), 6811-6817 doi:10.1016/j.biomaterials.2005.04.063

2. Seo KS, Yoon JW, Na KH, Bae EJ, Woo JG, Lee SH, et al. Evaluation of process efficiency and bioequivalence of biosimilar recombinant human chorionic gonadotropin (rhCG). *BioDrugs* (2011) 25(2), 115-127 doi:10.2165/11589430-000000000-00000

3. Takai T, Takaoka M, Yasueda H, Okumura K and Ogawa H. Dilution method to refold bacterially expressed recombinant Der f 2 and Der p 2 to exhibit the secondary structure and histamine-releasing activity of natural allergens. *Int Arch Allergy Immunol* (2005) 137(1), 1-8 doi:10.1159/000084607
